# Protocol for the Proactive Or Reactive Telephone Smoking CeSsation Support (PORTSSS) trial

**DOI:** 10.1186/1745-6215-10-26

**Published:** 2009-04-28

**Authors:** Tim Coleman, Andy McEwen, Linda Bauld, Janet Ferguson, Paula Lorgelly, Sarah Lewis

**Affiliations:** 1 Division of Primary Care and UK Centre for Tobacco Control Studies, University of Nottingham, Nottingham, UK; 2 Cancer Research UK Health Behaviour Research Centre, Department of Epidemiology & Public Health, University College of London, London, UK; 3 Department of Social and Policy Sciences and UK Centre for Tobacco Control Studies, University of Bath, Bath, UK; 4 Section of Public Health and Health Policy, University of Glasgow, Glasgow, UK; 5 Division of Epidemiology & Public Health and UK Centre for Tobacco Control Studies, University of Nottingham, Nottingham, UK

## Abstract

**Background:**

Telephone quit lines are accessible to many smokers and are used to engage motivated smokers to make quit attempts. Smoking cessation counselling provided via telephone can either be reactive (i.e. primarily involving the provision of evidence-based information), or proactive (i.e. primarily involving repeated, sequenced calls from and interaction with trained cessation counsellors). Some studies have found proactive telephone counselling more effective and this trial will investigate whether or not proactive telephone support for smoking cessation, delivered through the National Health Service (NHS) Smoking Helpline is more effective or cost-effective than reactive support. It will also investigate whether or not providing nicotine replacement therapy (NRT), in addition to telephone counselling, has an adjunctive impact on smoking cessation rates and whether or not this is cost effective.

**Methods:**

This will be a parallel group, factorial design RCT, conducted through the English national NHS Smoking Helpline which is run from headquarters in Glasgow. Participants will be smokers who call the helpline from any location in England and who wish to stop smoking. If 644 participants are recruited to four equally-sized trial groups (total sample size = 2576), the trial will have 90% power for detecting a treatment effect (Odds Ratio) of 1.5 for each of the two interventions: i) proactive versus reactive support and ii) the offer of NRT versus no offer. The primary outcome measure for the study is self-reported, prolonged abstinence from smoking for at least six months following an agreed quit date. A concurrent health economic evaluation will investigate the cost effectiveness of the two interventions when delivered via a telephone helpline.

**Discussion:**

The *PORTSSS *trial will provide high quality evidence to determine the most appropriate kind of counselling which should be provided via the NHS Smoking Helpline and also whether or not an additional offer of cost-free NRT is effective and cost effective for smoking cessation.

**Trial Registration:**

(clinicaltrials.gov)**: **NCT00775944

## Background

Smoking remains a massive public health problem in the UK and, for example, is the single most important avoidable cause of cancer, responsible for an estimated 45,000 cancer deaths, and 110,000 hospital admissions in the UK each year.[1] Smoking prevalence is strongly associated with social disadvantage[1,2] and is the largest identified cause of social inequalities in health.[2] Consequently, any reductions in smoking prevalence will result in substantial population health gain. Comprehensive, population-level tobacco control measures, such as the California Tobacco Control Program are likely to have the greatest impact on smoking prevalence[3] because the interventions included in this kind of programme reach vast numbers of smokers; presumably prompting them to attempt cessation. However, the provision of effective smoking cessation interventions to smokers making quit attempts remains important, because smokers have a much higher probability of achieving permanent abstinence when they benefit from evidence-based smoking cessation interventions to assist their efforts to stop.

In England, smokers can be supported during quit attempts by general practitioners: GPs' brief advice against smoking is effective[4] and GPs can prescribe proven nicotine addiction treatments like nicotine replacement therapy (NRT)[5], bupropion[6] and varenicline.[7] Additionally, smokers who are particularly motivated to stop can attend NHS Stop Smoking Services (NHSSS)[8] where they can access effective group[9] or individual[10] behavioural support. Many NHSSS issue vouchers for NRT which smokers can then redeem from pharmacies and others issue NRT directly to patients, without prescription from a doctor, via Patient Group Directives. Unfortunately, opportunities to deliver effective interventions to smokers are frequently missed in primary care[11] and, from a population perspective, relatively few smokers actually attend NHSSS.[12] It is logical, therefore, to develop alternative methods for supporting smokers in quit attempts and the provision of telephone counselling support to smokers who contact quitlines is one such method.[13]

Clearly, the provision of proactive telephone counselling could be an effective, additional smoking cessation intervention to use within an overall programme of tobacco control measures. The considerable accessibility and 'reach' of a national quitline delivering such counselling should have a demonstrable impact on callers' cessation rates and could even have an impact on national smoking prevalence (though this should not be an expected outcome of introducing one). Additionally, the effectiveness of quitlines for promoting smoking cessation would be enhanced if quitline callers could be encouraged to use established, effective and safe smoking cessation treatments like NRT. In England, a reactive support package called the 'Together Programme' is provided to smokers who contact a national quitline called the NHS Smoking Helpline and, in 2008, proactive support is to be added as an option to help quitline callers to stop smoking. Additionally, to augment the effectiveness of counselling alone, the quitline plans to begin offering cost-free NRT to callers for whom this is appropriate.[14] NRT is particularly appropriate for use in this way as 100+ trials conducted world wide have demonstrated its undisputed safety and efficacy, even when used without guidance and advice from a health professional.[15] Consequently, some NRT formulations have a licence for general sale (GSL) in the UK, so they can be retailed outside of pharmacies.

This trial will, therefore, determine for smokers who call the NHS Smoking Helpline, whether or not proactive telephone counselling is more effective or cost effective for smoking cessation when compared to reactive counselling. Additionally, it will investigate whether or not offering quitline callers a voucher for a cost free supply of NRT, compared to merely advising them to obtain drug treatment for nicotine addiction augments quit rates from behavioural interventions alone. The influence of economic deprivation on the effectiveness of interventions will be investigated to assess the potential for trial interventions to impact on smoking-related health inequalities.

### Aim

To determine whether or not proactive telephone support for smoking cessation delivered to quitline callers is more effective and cost effective than standard 'reactive' provision and whether or not the offer of a voucher for free NRT has any additional impact on smoking cessation rates achieved by behavioural interventions.

### Objectives

1. To compare, amongst motivated callers to the NHS Smoking Helpline, the effectiveness and cost effectiveness of reactive and proactive quitline care for smoking cessation.

2. To compare, amongst motivated callers to the NHS Smoking Helpline, the effectiveness and cost effectiveness of, in addition to telephone behavioural counselling, two strategies for making NRT available: i) offering a voucher for free provision and ii) advising nicotine addiction treatment.

3. To investigate whether or not the provision of telephone counselling for smoking cessation via the NHS Smoking Helpline is more or less effective for disadvantaged smokers.

4. To document uptake of telephone counselling and offered NRT in trial arms.

## Methods/Design

**Figure 1:**
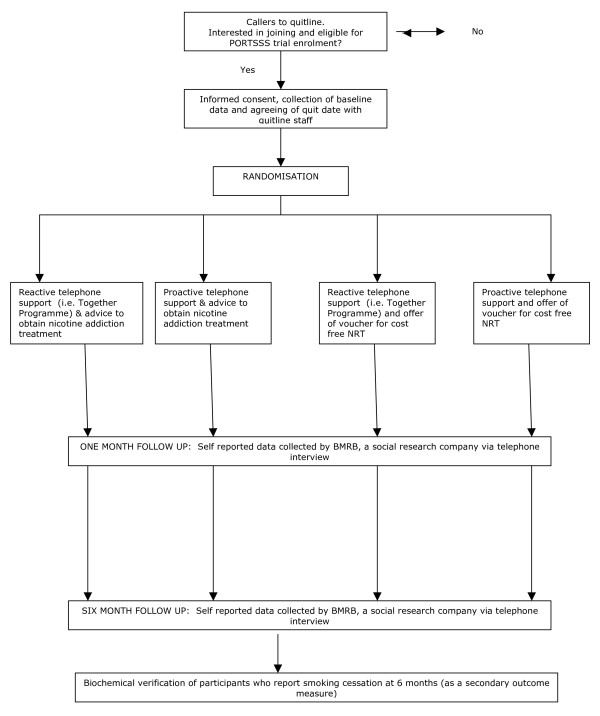
**PORTSSS Trial Participant Flow Chart**.

Ethical approval for the study was granted on behalf of the National Research Ethics Service by Leicestershire, Northamptonshire & Rutland Research Ethics Committee. This is a four group, parallel, factorial randomised controlled trial with investigators blinded to participants' treatment allocation. Figure 1 summarises the trial design.

### Duration of the Trial and Participant Involvement

The trial will recruit participants for approximately 6 months, with the final length of the recruitment period dependant on the actual recruitment rate. The trial will close after the final follow up of the last recruit to the study at 6 months after (s)he is enrolled. The total duration of the study is, thus, approximately one year.

Enrolled participants will agree a quit date for stopping smoking and will be allocated to one of the four trial groups described above/below. More complete details of trial interventions follow later:

1. Usual telephone support with smoking cessation (the 'Together Programme')

2. As per group 1 plus additional proactive telephone support

3. As per group 1 plus the offer of a voucher for free NRT

4. As per group 2, plus the offer of a voucher for free NRT

Follow up data will be collected during telephone interviews at one and six months after the agreed quit date. Participants who report smoking cessation at final follow up (estimated 10% in total) will be asked to provide an exhaled air carbon monoxide (CO) reading for biochemical verification of smoking status.

### Primary Outcome Measure

Self-reported, prolonged abstinence from smoking between a quit date and 6 months afterwards.

To demonstrate prolonged**abstinence, a participant must report not smoking at the 6 month point, but may admit to minor lapses, provided that they have smoked no more than 5 cigarettes since their agreed quit date.[16]

### Secondary Outcome Measures

1. Self-reported, prolonged abstinence from smoking between a quit date and 6 months afterwards with biochemical validation by exhaled carbon monoxide measurement at the 6 month point.

2. Self-reported, abstinence from smoking between a quit date and 3 days afterwards (NB: this outcome is ascertained by helpline counsellors, other outcomes ascertained by a market research company)

3. Self-reported point prevalence abstinence from smoking for at least 7 days, ascertained at 6 months with biochemical validation

4. Self-reported point prevalence abstinence from smoking for at least 7 days, ascertained at 6 months

5. Self-reported abstinence from smoking for at least three months, ascertained at 6 months

6. Self-reported prolonged abstinence from smoking between a quit date and 1 month

7. Self-reported point prevalence abstinence from smoking for at least 7 days, ascertained at 1 months

8. Number of unsuccessful quit attempts lasting > 24 hrs, reported at 6 months

#### Non smoking-related outcomes

Health status at 6 months (EQ5D)[17]

Use of other NHS smoking cessation interventions (e.g. uptake of NHS Stop Smoking Services, use of other NRT obtained from GP etc.)

#### Process measures/measures of intervention uptake

1. Number of successful counselling calls made to participants

2. Number (percentage) smokers who accept offer of NRT vouchers

3. Number (percentage) smokers who are sent NRT from pharmacy after receipt of vouchers

4. Number (percentage) smokers who report using NRT

### Recruitment

The NHS Smoking Helpline is available to all smokers who reside in England. We aim to inform all smokers who call the NHS Smoking Helpline about the trial and to give more information about it to those who express an interest in participation, so that they can make an informed decision. Web-hosted and print media information which briefly informs people about the trial will be designed to accompany existing information about the NHS Smoking Helpline. This information will inform the reader that the NHS Smoking Helpline is experimenting with ways of providing support to smokers. Web based information will include a patient information sheet that smokers will be invited to download and read, prior to calling the NHS Smoking Helpline.

When smokers telephone the NHS Smoking Helpline, their call is initially taken by a non-counsellor telephonist employed by Broadsystems, a company based in Bristol UK. Broadsystems staff will not routinely give further information about the trial to callers but will follow their usual procedure which for smokers who want help with stopping smoking, involves a mid-call transfer to speak with one of a team of specialist smoking cessation advisors who work for a company called Essentia. Any callers who ask Broadsystems' telephonists for further information about the trial will also be referred on to Essentia and specially-trained Essentia smoking cessation advisors will be responsible for obtaining informed consent from trial participants. Essentia staff will use a scripted protocol to verbally-inform interested callers about relevant aspects of the trial and to invite them to participate. Any caller who, after discussion, is not interested in the notion of trial participation will receive normal NHS Smoking Helpline care, which might involve registering for the Together Programme (TP).

Completion of the scripted consent protocol will be sequential, such that counsellors must confirm that initial issues have been completely addressed before moving onto subsequent ones. At the end of discussion about trial enrolment, participants will be aware that their participation is voluntary, that if they decline to participate, their care will not be affected and that they can withdraw from the study at any time. Participants will also be made aware that, in the event of their withdrawal from the trial, data already collected would be used in analysis unless specific permission/consent to use these data is also withdrawn. All of these issues will be thoroughly dealt with by the patient information sheet that can be accessed prior to trial enrolment and which will be sent to all smokers who decide to join the trial (see later).

### Inclusion criteria

Participants are over 16 and will need to agree to i) receive counselling ii) to set a quit within four weeks and iii) consent to follow up.

### Exclusion criteria

Telephonists will not enrol potential participants who are not capable of giving informed consent or who have not got access to a phone contact number to which calls can be made by Essentia staff. The language of the usual Essentia interview is English and it will not be possible to enrol participants who cannot understand this.

### Expected duration of participant participation

In both arms of the trial, participants will be offered telephone counselling for up to 3 weeks after their agreed quit date and participants who redeem NRT using vouchers will be able to use this for up to 6 weeks.

### Removal of participants from therapy or assessments

As is usual with this type of research, after randomisation participants may choose not to use trial interventions (i.e. they may ignore telephone calls or written materials received in the post). Also a substantial proportion of participants who do use trial interventions will not use these as per protocol (e.g. they may decide not to participate in a complete 'course' of phone calls). Such participants will still be included in the final analysis which uses an intention to treat approach.

There is no risk to participants from behavioural counselling. NRT patches are available over the counter (OTC) and, in 'NRT arms' a voucher for an OTC preparation will be provided to quitline callers who want this and who report no contraindications to their use. Upon voucher redemption, NRT patches will be provided in the same packaging as they are sold OTC and, in addition to any advice on NRT that counselling interventions may include, participants will be asked to read directions for NRT use that are included with packaged OTC NRT. Participants may resolve any questions that they have about NRT use by discussion with a helpline counsellor or via NRT patient information sheets and in the unlikely event of residual uncertainty, they will be advised to discuss this with their GP. The use of vouchers for NRT, issued by smoking cessation counsellors mimics current clinical practice, as NRT vouchers are distributed by NHS Stop Smoking Service advisors in many parts of England. It is anticipated that there will be no circumstances in which the trial investigators will need to instruct participants to discontinue NRT treatment, though counsellors advising participants may need to recommend this. Additionally, participants themselves may decide to discontinue treatment after reading NRT usage instructions (e.g. if a rash develops). In the rare eventuality of a major problem (e.g. with the quality of supplied NRT), the research team would be able to contact participants who had redeemed vouchers and advise them. It should be noted that, in this unlikely eventuality, participants would be easier to identify and contact than members of the public who had bought NRT OTC.

Participants may be withdrawn from the trial at their own request and, if they do so, will be made aware that this will not affect their future care. Participants will also be made aware, should they withdraw, the data collected to date would only be erased and not used in the final analysis, if they were to specifically request this.

### Informed consent

All staff who obtain participant consent for the trial will receive appropriate training and will be named in a trial delegation log and, hence, authorised to do so. The scripted protocol that trained Essentia staff use (see section 'Recruitment', above), will ensure all important trial issues which might concern the participant are discussed and a copy of the PIS will be sent to participants. Verbal consent *only*******will be required and, to participate, quitline callers must give explicit verbal consent to:

• permit routine data collected by Essentia to be used for research

• telephone follow up by BMRB Social Research (a commercial survey company)

• agree to a quit date with the Essentia counsellor/telephonist, before being randomly allocated to one of the trial treatment arms

• accept telephone calls from Essentia smoking cessation counsellors at a number of their choice and, if necessary, allow counsellors to leave messages concerning the reason for calling (i.e. smoking cessation support) at this number

• having their personal details transferred by secure electronic methods between Essentia, BMRB, a pharmacy and the University of Nottingham research team.

• potentially being asked to give an exhaled breath air sample to a BMRB fieldworker

Immediately after the scripted consent protocol has been completed and participant's consent obtained, the Essentia counsellor who has conducted this process will generate three hard copies of a trial consent form. Database restrictions will not permit form generation until the consent protocol has been fully completed. Consent forms will be automatically populated with the date and time that consent was taken and also with an identifier or initials of the Essentia staff who obtained consent. This will provide the trial manager or any other auditor with a clear audit trail of who generated the consent form and will also provide, if ever deemed necessary, the potential to cross reference time of consent with that of the participants' call. Consent forms will also include participants' names, addresses and telephone numbers. Copies will be: i) received by the *PORTSSS *trial manager for research governance purposes ii) stored within Essentia, so there is a record of callers enrolled into the trial and iii) sent to participants. The copy sent to participants will be accompanied by a participant information sheet (PIS), with a letter encouraging participants to read the PIS and think about their decision to join the trial. Documentation will include details of a contact point for participants who have questions about trial participation.

The method of obtaining consent will allow this trial to mimic normal quitline practice as quitline interventions are usually delivered to callers during a single telephone call at the time of their first contact. The proposed approach is justifiable, given the minimal risk to trial participants posed by trial interventions and the fact that, in other contexts, these have all been demonstrated as effective for smoking cessation. The relatively light burden of tasks imposed on participants and the fact that they will effectively choose how much time and energy they devote to trial participation also adds to the justifiability of the consent process.

Most importantly, it would be very difficult, and perhaps impossible, to answer the questions posed by the trial without using the outlined approach to obtaining informed consent. If consent forms signed by participants were required, this would undoubtedly result in many callers verbally agreeing to consent, but not subsequently returning signed confirmation, despite still consenting to participate. In this circumstance recruiting to a trial would be difficult and providing robust experimental data about the effectiveness of quitline interventions would be a challenge. Additionally, the external validity of trial findings would be severely compromised and one would not be able to say whether or not trial findings were actually relevant to quitline practice. By obtaining verbal consent and following this up with written information, some participants may decide after the initial recruitment discussion, that, on balance, they do not wish to participate further. As mentioned above, these non-participants will be given information about how to contact the trial team to withdraw consent, but even if they do not exercise this option, they will still be able to ignore telephone or postal contact from Essentia (delivering interventions) or the trial team/BMRB (conducting follow up).

### Randomization and Blinding

#### Level of blinding

Researchers conducting participants' follow up, the trial manager and the trial management team will be blind to treatment allocations. The trial statistician will also be blinded to treatment allocation whilst the trial is progressing, but, as is necessary with a factorial designs, will need to identify treatment combinations during data analysis. Trial participants will be aware of interventions that they receive and counsellors giving trial treatments cannot be blinded to these.

#### Randomisation process

All smokers who register with the 'Together Programme' will be eligible for trial enrolment and Essentia counsellors will randomise callers to one of four treatment groups after obtaining their informed consent. A computer programme will generate a random number sequence and, after randomisation, participants will be issued with a unique trial number and a code which corresponds to their treatment and the first session of the allocated treatment will be delivered. Any data that are exported for use by the research team will have the treatment allocation codes stripped out to maintain blinding. A key for the numerical code will be held by the trial statistician who will conduct the sole statistical analysis of trial data after the trial has ended.

### Baseline Measures

Consent will be obtained to use the following data items which are routinely-obtained from all smokers who register with the Together Programme:

birth date, gender postcode, employment status, ethnic group, pregnancy, eligibility for free prescriptions, previous use of treatments for nicotine addiction, strength of nicotine addiction as measured by the 'heaviness of smoking index'[18] and quit attempts in the previous year.

Additionally, health status measured by EQ5D[17] will be ascertained and participants will agree a quit date for stopping smoking which is within four weeks. It is important that the quit date is agreed before randomisation because in a similar study, setting a quit date was not a criterion for trial entry and approximately 43% of those enrolled did not subsequently set one.[19] By requiring participants to set quit dates, we will ensure that only the most motivated smokers enrol into the trial and each participant will have a definite event from which subsequent counselling sessions and research follow ups can be timed.

### Trial Groups

After randomisation, Essentia staff will be automatically guided through the correct intervention for each participant via four on-screen templates. The Essentia database will be configured so that only the template which corresponds to the appropriate treatment allocation can be used. Participants will be randomly allocated to either usual care (i.e. control group, called the 'Together Programme') which comprises reactive counselling or proactive counselling (intervention 1) as described below. Within these two groups there will also be random allocation of smokers to either being offered advice to obtain nicotine addiction treatment (i.e. NRT, Varenicline or Bupropion) from their general practitioner or local NHSSSs (control) or an offer of a voucher which can be redeemed at a pharmacy for a free supply of NRT (intervention 2). The aim is to have four trial groups of equal size.

### Control – Together Programme

Usual Together Programme care involves offering smokers support with smoking cessation via telephone, email, written materials and/or text message and, if appropriate, advising them to seek further support (potentially including nicotine addiction therapies) from their local NHSSS or general practitioner. After their first session, participants will be contacted by telephone (unless they prefer email or text message) on their quit dates and at two days and three weeks afterwards with brief motivational messages.

### Intervention 1 – Proactive telephone counselling

Smokers will be offered usual "Together Programme" written materials and modified email and text messages, plus a programme of proactive telephone counselling following an agreed protocol, delivered by specially-trained Essentia counsellors. After their initial counselling session, participants will receive another call for counselling prior to their quit date and also on the day that they stop smoking itself. Subsequently participants will be eligible to receive up to 4 further telephone counselling sessions in the 3 weeks after their quit date. Earlier sessions will help smokers to focus on and prepare for quitting and later ones will focus on remaining smoke-free and preventing relapse. This call pattern is based on a previously-trialled, US quitline calls schedule and the weekly contacts made after smokers' quit dates follow a similar frequency to that used for face-to-face counselling by many NHSSSs. During counselling and, if appropriate, smokers will be advised to obtain further support (including, potentially, nicotine addiction therapies) from their local NHSSSs or general practitioners. Dr Andy McEwen (UCL – a protocol author) will develop the intervention protocol and train counsellors in proactive telephone counselling.

### Intervention 2 – Provision of voucher for free NRT

During discussion of nicotine addiction treatments, callers will be offered a voucher which can be redeemed at a pharmacy for a cost free supply of NRT. Not all smokers will choose to accept the voucher. For example, some smokers might have previously used NRT, so that they do not believe using this again is likely to be effective. Others might prefer to try different treatments for nicotine addiction which can be obtained from other sources.

For smokers who choose to accept the offer of an NRT voucher, the Essentia counsellor will run through with them a checklist of cautions to NRT. This checklist will be identical to that which is in the product information that accompanies the over the counter (OTC) formulation of NRT used in the trial. If callers report uncertainties about any of the listed cautions applying to them, a voucher will not be issued and they will be advised to discuss the potential use of NRT with their GP. Vouchers will be generated electronically by the Essentia counsellor and sent to the pharmacy team responsible for voucher redemption and participants will be given a freephone number to call for voucher redemption. Confirmation of this number will sent by email, text or paper mail according to participants' preferences.

A 15 mg/16 hr transdermal nicotine patch which is available over the counter (OTC) and, hence, on the UK 'General Sales List' will be used and there are no specific recommendations regarding their purchase or storage. Although smokers in the UK can purchase such medication OTC, we propose that voucher redemption is overseen by a pharmacist to provide reassurance about trial safety. NRT will be stored securely within a Glasgow pharmacy and a secure system for recording i) when NRT has been issued against any one voucher and ii) the batch number of NRT sent to individuals will be used. The pharmacy team will be part of the NHS Greater Glasgow and Clyde's Public Heath Pharmacy Department.

Participants will be required to telephone the pharmacy team to redeem their voucher and obtain NRT and they will not be directly sent NRT without making such a request. Participants' vouchers will make them eligible for up to 6 weeks cost-free NRT treatment, supplied in batches. All NRT packaging and instructions will be identical to that which has been approved for retail sale on a General Sales License outside of pharmacies

In all trial groups, if smokers have not tried to stop on their agreed quit date and they still wish to stop smoking, a new (i.e. second) quit date will be agreed and the timing of follow-ups will be tied to this point but no further re-negotiation of quit dates will be permitted.

### Compliance

This is a pragmatic trial, testing the delivery of interventions in routine clinical use, using an intention to treat analysis. In such studies it is usual for participants to have variable compliance with interventions as their level of engagement with these is self selected. Consequently, uptake rates for interventions (e.g. the proportion of subjects who redeem NRT vouchers) are amongst study outcomes.

### Frequency and duration of follow up

Interim follow up will occur at one month and final follow up is at six months which is consistent with agreed standards for the measurement of smoking cessation outcomes in trials.[16] BMRB Social Research, a social research company, will contact trial participants to obtain follow up data. All participants will be asked about smoking outcomes and health status (EQ5D) and up to 10 attempts will be made to contact participants before they are categorised as 'lost to follow up'. Exhaled carbon monoxide readings will be used to assess smoking status amongst those smokers who report either i) continuous abstinence from smoking cessation between their quit date and 6 months, ii) 7 day (or longer) point prevalence abstinence from smoking at 6 months. BMRB staff will visit those smokers who report smoking cessation and obtain exhaled CO samples. A cut off point of 10 ppm of CO[16] in exhaled air will be used to differentiate between smokers and non-smokers and three attempts at CO validation will be made before any participant is recorded as non-contactable.

### Methods for protecting against bias

It will be impossible for Essentia staff delivering trial interventions to predict the outcome of randomisation, so there should be no bias in the allocation of participants to trial groups. All staff collecting follow-up information will be blind to participants' treatment allocations. As participants cannot be blinded to their allocated intervention, information about trial interventions will be carefully worded to ensure that this does not contribute to higher drop out rates in any one trial arm.

## Statistical Methods

### Sample Size and Justification

Sample size has been calculated to ensure that the trial is adequately powered to detect meaningful differences in the primary outcome measure (self report) and also in the secondary outcome that involves biochemical validation of this. A Cochrane review which compares, for smokers who call quitlines, proactive telephone cessation counselling with very brief counselling and/or the provision of smoking cessation materials only shows that proactive counselling is effective (OR = 1.41, 95%CI 1.27 to 1.57)[13]. Furthermore, there is evidence that telephone counselling is slightly more effective for motivated smokers (i.e. those prepared to make a quit attempt) [OR (in favour of motivated) 1.64, 95%CI 1.41 to 1.92] [13]. For the PORTSSStrial, we will assume that proactive counselling (compared to reactive) will have a treatment effect with an OR of 1.5. Given that we intend to recruit only motivated smokers to this study, however, this is probably a conservative assumption. The impact of telephone counselling is likely to be increased by the offer of NRT vouchers; two non-randomised US studies have shown that offering NRT (or NRT vouchers) in addition to standard quitline increased cessation rates at 6 months by 80% and 77% respectively[14,20]. A further randomised controlled trial with a factorial design assessed the impact of three levels of intensity of telephone counselling and of the offer of NRT and found an effect of NRT (OR = 1.58) and no evidence of interaction between the effects of counselling intensity and offer of NRT [21]. Consequently, we will assume that the offer of NRT vouchers is also associated with a treatment effect with an OR of 1.5, i.e. that the size of this effect is similar to that for proactive compared to reactive support, and that there will be no interaction between the effects of these two interventions.

**Figure 2:**
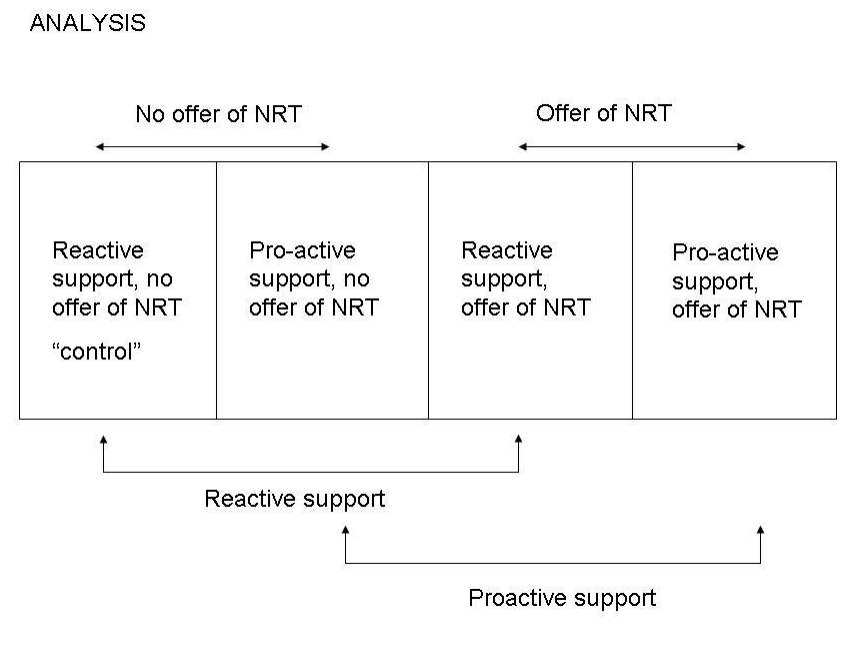
**Comparison Groups within PORTSSS Trial**.

Predicting control group (reactive support, no offer of NRT) quit rates for the PORTSSStrial is difficult because no previous UK trials have specifically enrolled motivated smokers (i.e. those who agree to make a quit attempt) and also previous trials have relied solely on self-report measures of smoking behaviour. Recruiting motivated smokers to PORTSSSis likely to increase quit rates at 6 months relative to previous UK trials, whereas using biochemical validation of smoking behaviour will probably diminish the number of cases for which primary outcome data is ascertained, apparently decreasing PORTSSS's quit rates. However, two recent UK trials with similar control group interventions to that proposed in PORTSSSreported, at 6 months, rates of prolonged abstinence from smoking for at least 3 months of 11.6%[19] and 9.0%[22], respectively. Consequently, we anticipate that in PORTSSS, self-reported prolonged abstinence between smokers' quit dates and their 6 month follow up will be 12% in the control group (reactive support, no offer of NRT). However, our experience shows us that up to 1/3 of participants who claim abstinence from smoking will not provide saliva samples at follow up, so we reduce the expected abstinence rate in the control group to 8%. In this factorial design, assuming no interaction between treatments, the comparison group for each treatment will comprise the combination of the control group (who receive neither treatment) and the group receiving only the other treatment (see Figure 2). For example, if the odds ratio for each treatment is 1.5 and the abstinence rate in the control is 8%, the comparison "group" would be expected to have an abstinence rate of 10% (i.e. the average of 8% and 12%). In the absence of interaction, we plan two a priori comparisons of the effect of each treatment versus the appropriate comparison group, using p < 0.05 as the level of statistical significance. Table 1 illustrates how variations in treatment effects and control group quit rates might affect the sample size required to have 80% power and 90% power (i.e. 90% chance of detecting a real effect caused by the trial intervention). Remember that in this table intervention and control groups are amalgamations of trial arms such that NRT versus no NRT and proactive versus reactive counselling are compared.

**Table 1:** Sample sizes with different treatment effects and control group quit rates

Control (reactive support, no offer of NRT) group quit rate at 6 months (%)	Comparison "group" quit rate at 6 months (%)	Intervention "group" quit rate at 6 months (%)	Odds ratio for each treatment effect (proactive vs reactive, NRT vs no NRT)	Power	Required sample size per group (total for 4 groups)
12%*	14%	18.6%	1.4	80%	536 (2142)

12%*	14.5%	20.3%	1.5	80%	355 (1420)

12%	14.5%	20.3%	1.5	90%	468 (1872)

8%**	9.5%	12.8%	1.4	80%	739 (2956)

8%**	**10%**	**14%**	**1.5**	**80%**	**489 (1956)**

8%**	**10%**	**14%**	**1.5**	**90%**	**644 (2576)**

* self report quit rates, ** biochemically validated quit rates

Final row figures (bold) show our most likely estimates for required sample sizes.

A further consideration is the increase in type 1 error arising as a result of including two primary treatment comparisons: proactive versus reactive counselling and NRT versus no NRT. A more conservative approach would adjust the p value to reflect the number of comparisons to be made i.e. take p = 0.025 (0.05/2). Table 2 shows the same power calculations as above using the more conservative p = 0.025.

**Table 2:** Sample sizes with different treatment effects and control group quit rates using conservative 'p' value (0.025)

Control (reactive support, no offer of NRT) group quit rate at 6 months (%)	Comparison "group" quit rate at 6 months (%)	Intervention "group" quit rate at 6 months (%)	Odds ratio for each treatment effect (proactive vs reactive, NRT vs no NRT)	Power	Required sample size per group (total for 4 groups)
12%*	14%	18.6%	1.4	80%	644 (2576)

12%*	14.5%	20.3%	1.5	80%	426 (1704)

12%	14.5%	20.3%	1.5	90%	550 (2200)

8%**	9.5%	12.8%	1.4	80%	888 (3552)

8%**	**10%**	**14%**	**1.5**	**80%**	**587 (2348)**

8%**	**10%**	**14%**	**1.5**	**90%**	**760 (3040)**

* self report quit rates, ** biochemically validated quit rates

Final row figures (bold) show our most likely estimates for required sample sizes.

To allow for this more conservative analysis we should aim to recruit 3040 participants for 90% power. However, if we aim to recruit 2576 we would have 90% power to detect these differences as significant using a 5% significance level, and at least 80% power to detect these differences as significant at the more conservative 2.5% significance level.

### Planned recruitment rate

It should be feasible to recruit 2576 smokers in approximately 6 months, though this can only be confirmed after recruitment begins. In the first 6 months of 2007, 13,418 smokers registered with the Together Programme and the trial will recruit from this pool of callers. In a previous trial, only 28% of quit line callers who wanted counselling also agreed to trial enrolment[19] and our necessary requirement that participants agree to set a quit date could further reduce enrolment and randomisation rates amongst Together Programme registrations. Without piloting, one cannot be entirely certain about the proportion of callers who will agree to trial enrolment, but it is likely that this will be sufficient for recruitment.

### Potential problems with compliance

Participants randomised to receive proactive telephone support may not actually answer or be available for many of the calls that are made to them. If this situation were to arise, then it could attenuate any differences between interventions delivered to the control and intervention groups, making it harder to detect an effect attributable to the proactive call protocol. Additionally, participants who register for the Together Programme are currently able to telephone smoking cessation advisors whenever they wish and if a large number make such calls were to be made, this could further reduce differences between interventions. Consequently, treatment protocols in the reactive and proactive arms of the trial will need to be clearly defined.

### Expected rate of loss to follow-up

Previous UK studies have experienced losses to follow up in the region of 30%[19,22] and we have planned for a similar rate in this study, despite hoping to improve upon this by using a specially-commissioned market research company for follow up.

### Statistical Analysis Methods

Primary analysis will be by intention to treat, presuming continued smoking in those lost to follow-up. We will initially look for evidence of interaction between the effects of the two treatment interventions (proactive versus reactive support, and offer of NRT voucher versus no voucher offer) using a test of interaction in a logistic regression model. We do not anticipate interaction and presuming there is none, we will compare those who were randomised to proactive support (i.e. the combination of two groups) with those randomised to reactive support, and those offered of NRT with all those not offered NRT, using two distinct chi-squared tests (see Figure 2 below).

We will compare baseline characteristics of intervention and usual care groups, adjusting for any baseline differences using multiple logistic regression. We will carry out a sensitivity analysis to determine the influence of how missing data at follow-up are handled on the study conclusions, varying the strength of association between smoking status and 'missingness' (i.e. whether data are missing), and allowing for individual, sampling and imputation variation using multiple imputation.[23] We will investigate whether or not socio-economic deprivation (as measured by Townsend's score) is associated with successful outcome by looking for effect modification using a test for interaction to establish whether the effect of each treatment is similar in more versus less deprived groups. The data will be analysed at the end of the study only; there is no need for interim analyses on safety grounds, and having interim analyses alters the chance of type 1 or type 2 error.

### Procedures for missing, unused and spurious data

The standard method for dealing with loss to follow-up in smoking cessation trials is to assume that those lost-to-follow are continuing to smoke.[16] However, we will also use methods of multiple imputation as suggested by Hedeker[23] to explore the effect of assuming alternative associations between 'missingness' and smoking status as explained above.

### Definition of populations analysed

As this is an intention to treat analysis, all participants who cannot be contacted at follow up are assumed to be still smoking and all randomised participants are included in all analyses for trial outcomes. We will look for effect modification by actual redemption of NRT voucher and by the intensity of proactive counselling received.

## Health Economic Analysis

The economic analysis will be presented separately for the within trial period (to summarise the observed evidence in relation to cost-effectiveness) and for a projected lifetime cost-effectiveness. Within trial analyses will be presented both to test the underlying hypotheses and to provide necessary parameter estimates for the lifetime cost-effectiveness model. Resource use and cost data collected and estimated within the trial will be used, together with the primary outcome measure (cessation), to produce an estimate of the incremental 'cost per quitter' (that is the additional cost of improving the quit rate). The incremental cost per quality adjusted life year (QALY) gained will also be estimated. It is likely that EQ5D will be insensitive to change, therefore, the EQ5D data will be more informative for the longer term modelling. Probabilistic sensitivity analysis will be undertaken to understand the uncertainty surrounding the cost effectiveness estimates[24].

A model, previously developed in a separate smoking cessation project [25,26], which describes the long term health benefits of quitting smoking in terms of QALYs saved and potential reduced costs to the health service, will then be analysed. Using this model, we will combine trial outcome data and previously determined cost data with estimates from the literature to extrapolate the cost effectiveness at six months to one year, and then to a lifetime. The lifetime analysis will produce estimates of cost per QALYs gained, thereby establishing the long term cost effectiveness of proactive versus reactive smoking cessation support, with or without the offer of NRT vouchers. Probabilistic sensitivity analysis will be used to characterise uncertainty in the parameters of the model driven by estimates obtained from the trial. Finally, measuring health status via EQ-5D will allow us to compare health-related quality of life for trial participants with population norms and allow us to undertake further sensitivity analysis within the long term model.

## List of abbreviations

RCT: andomised Controlled Trial; UK: United Kingdom; EQ5D: EuroQol 5 Dimensions; PIS: Patient Information Sheet; BMRB Social Research: British Market Research Bureau Social Research; UCL: University College London; GP: General Practitioner; CO: Carbon Monoxide

## Competing interests

Within the last five years Tim Coleman has undertaken consultancy work for Pierre Fabre Laboratories, France and also Johnson & Johnson. Both companies produce nicotine replacement therapy. Andy McEwen has received travel funding, honorariums and consultancy payments from manufacturers of smoking cessation products (Pfizer Ltd, Novartis and GSK Consumer Healthcare Ltd). He also receives payment for providing training to smoking cessation specialists and receives royalties from books on smoking cessation. Linda Bauld, Paula Lorgelly, Sarah Lewis and Janet Ferguson declare that they have no competing interests.

## Authors' contributions

All authors contributed to the creation of this manuscript and also to the design of the trial. Tim Coleman is the guarantor for this paper.
